# Modulation of Serum Protein Electrophoretic Pattern and Leukocyte Population in Horses Vaccinated against West Nile Virus

**DOI:** 10.3390/ani11020477

**Published:** 2021-02-11

**Authors:** Francesca Arfuso, Elisabetta Giudice, Simona Di Pietro, Giuseppe Piccione, Claudia Giannetto

**Affiliations:** Department of Veterinary Sciences, Polo Universitario dell’Annunziata, University of Messina, 98168 Messina, Italy; farfuso@unime.it (F.A.); egiudice@unime.it (E.G.); simona.dipietro@unime.it (S.D.P.); claudia.giannetto1@unime.it (C.G.)

**Keywords:** West Nile virus, equine, protein electrophoretic fractions, inactivated vaccine, hematological parameters, serum total proteins

## Abstract

**Simple Summary:**

Vaccination is the primary method of reducing the risk of West Nile virus (WNV) infection in horses but clinical disease is not fully prevented. The aim of this study was to evaluate the hematological parameters, including the leukocyte population and the serum protein electrophoretic pattern of horses subjected to two-dose vaccine administration with inactivated WNV. Vaccine-related changes in lymphocytes, neutrophils, monocytes, serum total proteins, α1-, α-2-, β- and γ-globulin fractions are found.

**Abstract:**

This study aimed to evaluate the hematological and serum protein electrophoretic profiles of horses after inactivated West Nile virus (WNV) vaccine administration. Blood samples were collected from 10 horses before (T0), after 24 h, 48 h, 72 h, 1 week, 2 weeks and 3 weeks (T1_I_, T2_I_, T3_I_, T4_I_, T5_I_ and T6_I_) from the first WNV vaccine-dose administration, before the vaccine-booster (TPRE_II_), and after 24 h, 48 h, 72 h, 1 week, 2 weeks and 3 weeks (T1_I I_, T2_II_, T3_II_, T4_II_, T5_II_, T6_II_) from the WNV vaccine-booster. There was a significant increase in lymphocytes and a decrease in neutrophils after both the first vaccine-dose and vaccine-booster administration compared to the baseline values (*p* < 0.01). Monocytes showed higher values after 72 h, 1 week and 2 weeks from the vaccine-booster (*p* < 0.01). Higher serum total protein values were found in horses after both the first vaccine-dose and booster administration (*p* < 0.05). α1-lobulins increased after the vaccine-booster with the highest levels measured at T4_II_ (*p* < 0.05); α-2- and β-globulin fractions increased throughout the post-vaccine period compared to the baseline values (*p* < 0.05); and higher γ-globulin values were found before the vaccine-booster (TPRE_II_) and after 24 h, 72 h and 3 weeks from the vaccine-booster (T1_II_, T3_II_ and T6_II_). The findings allow us to conclude that the WNV vaccine used in the current study does not alter the overall hemogram picture of horses although it is associated with modulation of leukocyte populations and the serum protein electrophoretic pattern.

## 1. Introduction

The neurotropic flavivirus, West Nile virus (WNV) is a viral zoonotic disease that is considered a re-emerging public health challenge in European countries [1].

Since the beginning of the 2020 transmission season, European Union Member States have reported 316 human cases of WNV infection, including 37 deaths, and 185 outbreaks among equids [1,2]. Historically, the Mediterranean Basin is a crossroad for trade, transport, and migration. As a result, countries surrounding the Mediterranean Sea share common public health threats, and vector-borne diseases, in particular, mosquito-borne viral diseases are prime candidates as re-emerging diseases that are likely to spread across the area.

WNV infection was first diagnosed in horses in the United States in 1999 and is now an important consideration in the differential diagnosis of horses presenting with signs of neurologic disease in all areas of North America. West Nile virus, a flavivirus, was first identified as a cause of infection and fatal encephalomyelitis (inflammation of the spinal cord and brain) in horses and people in Egypt, Uganda and France in the early 1960s. Epizootics of disease in horses have occurred in Morocco in 1996, Italy in 1998, France in 2000, and the United States from 1999 to the present. West Nile virus is now considered to be endemic in all areas of North America [3,4,5].

In Italy, a significantly higher abundance of the vector of WNV has been recorded in warmer and less rainy conditions [2]. These conditions cause virus spill over outside the sylvatic cycle, to humans and/or equids. Most of the human and equine cases (98%, 289/294 and 95%, 226/238, respectively) in Italy occurred in the northern part of the country from 2013 to 2017, where the ecological conditions are most suitable for WNV transmission [3]. WNV transmission occurs primarily from April to November, when competent vectors are active and abundant. Mosquitoes, primarily Culex genus, serve as vectors of WNV and wild birds serve as reservoir hosts. Equids and humans are dead-end hosts [4,5]. WNV is closely related to other human pathogens such as Japanese encephalitis, Saint Louis encephalitis, tick-borne encephalitis, yellow fever (YFV) and dengue viruses. Humans infected with WNV develop a febrile illness that can progress to meningitis, encephalitis or acute flaccid paralysis. The elderly and immune-compromised are at greatest risk for severe encephalitic disease [1]. The majority of human cases remain asymptomatic; about 20% of infected individuals develop febrile illness and less than 1% develop severe neurological symptoms [6]. In comparison, 10% of infected equids develop neurological symptoms with different levels of severity [7]. 

The incubation period in horses is estimated to be 3–15 days and recovery is within 5–15 days. The mortality rate in horses with neurological symptoms may be as high as 38.00–57.10% [8]. Horses are not amplifying hosts as viraemia is low and transitory. Humoral immunity is a critical aspect of protection against flavivirus infection [9,10]. It is necessary for the initial containment and clearance of infection, as well as for subsequent protection against reinfection [11,12,13]. Eliciting a protective antibody response is a primary goal in the development of safe and effective vaccines [11].

Studies in animal models indicate that both innate and adaptive immune responses are required to protect against primary infection by virulent strains of WNV. Type I (α/β) and type II (γ) interferon, γδ T cell activation, an early neutralizing IgM response, and CD4+ and CD8+ T cells all contribute to and orchestrate the control and clearance of WNV from peripheral and CNS tissues (reviewed in [14]). The induction of high-titer WNV-specific and neutralizing antibodies after infection has been assumed as a primary mechanism of control during secondary challenge. Indeed, passive transfer of immune serum, monoclonal antibodies or polyclonal antibodies protects rodents from lethal primary WNV infection. Adaptive immunity and immune memory are modulated by numerous factors and mediators in early innate immunity. Although the importance of this regulation is well documented, the role of the acute phase response (APR) is still unclear. Understanding the function of acute phase proteins and their changing levels in response to immunization is of great value for vaccine design, development and administration. Vaccination has been shown to cause an inflammatory response, which results in homeostatic changes including changes in renal and hepatic functions [15,16]. One major homeostatic change that takes place in response to inflammation is the APR, during which the hepatic synthesis of albumin is reduced in favor of synthesis of acute phase proteins such as serum amyloid A (SAA) and fibrinogen [16]. In response to vaccination, acute phase proteins, can be used as reliable biomarkers for predicting the immune memory and vaccine efficacy [16].

A survey carried out on West Nile virus-seropositive blood donors has suggested that infection with WNV elicits a strong and durable antibody response with evidence of stable high-titer neutralizing antibodies even as far out as five years [17]. This indicates that subjects who have been infected with the virus may well have long-lasting protection from reinfection, and therefore, emphasizes that the development of vaccines that mimic natural infection could hopefully be expected to provide similar enduring immunity. It is clear that vaccine may be an important tool in controlling WNV infection during a natural outbreak or under conditions in which a rapid onset of protection is required [18,19]. To date, no vaccine is available for human, whereas a vaccine consisting of inactivated WNV strain, VM-2, was licensed in 2008 for use in horses in the EU by the European Medicines Agency (EMA). The vaccine is licensed for horses over six months of age [20]. Killed vaccines are considered relatively safe because the virus cannot replicate and cause clinical disease. The monitoring of several aspects of the physiological response of vaccinated animals is worthy of investigation. Indeed, complementary assessments are important to evaluate the strength of immunogenic stimulation. The quantitative assessment of the hemogram is important and has been used in many clinical situations, including infectious and traumatic diseases, surgery, chemotherapy and radiotherapy patients [21]. Another important test is protein electrophoresis, which evaluates the approximate concentration of different serum proteins including markers of inflammation and immunity [22].

In view of the above considerations, the aim of this study was to evaluate the hematological parameters including leukocyte population and the serum protein electrophoretic pattern of horses subjected to two-dose vaccine administration with inactivated WNV. The results could improve the current knowledge in this field and may provide valuable information of the status of the immune response as well as possible post-vaccine complications in WNV vaccinated-horses. 

## 2. Materials and Methods 

### 2.1. Animals and Experimental Design

Protocols for animal husbandry and experimentation were reviewed and approved in accordance with the standards recommended by the Guide for the Care and Use of Laboratory Animals and Directive 2010/63/EU for animal experiments.

Ten Italian saddle gelding horses (8–11 years old) with a mean body weight of 500 ± 25 kg from the same horse training center were enrolled in this study, which was carried out in December in Sicily, Italy (38°00′49″N 15°25′18″E, 80 m above sea level) with the owner’s informed consent. Before starting the study, horses were subjected to clinical examination, routine hematology and biochemistry analyses and only healthy subjects were used. All animals were housed in individual boxes (3.50 × 3.50 m) under a natural photoperiod and indoor temperatures. Thermal and hygrometric records were carried out inside the box for the whole study by means of a data logger (Gemini, Chichester, UK), and they followed the normal seasonal pattern for the location. The horses were fed three times a day (07:00, 12:00, and 19:00) with good-quality hay and concentrate (total food amount of about 2.5% of the horse body weight, forage:concentrate ratio 70:30). Water was available ad libitum. 

Each animal enrolled in the study was subjected to West Nile Disease vaccination with the free choice of the owners. Two doses of a vaccine that contains inactivated (killed) VM-2 strain West Nile virus, available as a 1 mL emulsion for intramuscular injection (Duvaxyn^®^ WNV, Zoetis, Italy), were given at four weeks later according to manufacturer’s recommendation. Vaccinated animals were checked by a veterinarian throughout and after the vaccination procedure.

### 2.2. Blood Sampling Procedures

Blood samples were collected from each horse by means of jugular venipuncture in vacutainer tubes containing EDTA and in tubes without anticoagulant agent (Terumo Corporation, Tokyo, Japan) at 14 time points: before the beginning of the vaccination protocol (before the first WNV vaccine-dose administration) (T0); after 24 h, 48 h, 72 h, 1 week, 2 weeks and 3 weeks from the first WNV vaccine-dose (T1_I_,T2_I_, T3_I_, T4_I_, T5_I_ and T6_I_); before the WNV vaccine-booster administration (TPRE_II_); and after 24 h, 48 h, 72 h, 1 week, 2 weeks and 3 weeks from the WNV vaccine-booster (T1_II_,T2_II_, T3_II_, T4_II_, T5_II_, T6_II_). Each blood sampling was performed at the same hour of the day in order to exclude the circadian influence on studied parameters [23,24].

### 2.3. Hematological Analysis

EDTA whole blood samples were processed in the laboratory within 2 h by means of an automated hematology analyzer (HeCo Vet C; SEAC, Florence, Italy) for the evaluation of complete blood count (CBC) including white blood cells (WBC), red blood cells (RBC), hematocrit (Hct), hemoglobin (Hb), mean corpuscular hemoglobin (MCH), mean corpuscular hemoglobin concentration, mean corpuscular volume (MCV) and platelets (PLT). Moreover a manual analysis was performed on all whole blood samples in order to perform leukocyte identification and counting. Specifically, two peripheral blood smears were performed for each sample, and after air drying, the obtained slides were stained through Dif-Stain kit (Titolchimica srl, Rome, Italy). The same laboratory professional later performed the microscopic analysis of blood films by using an optical microscope (Nikon Eclipse e200; Nikon Instruments Europe BV, Amsterdam, The Netherlands). A manual 100-cell differential count was performed on each blood film. For each animal, the leukocyte differential count was calculated by averaging the data recorded from each blood film of the same sample. Blood samples collected into vacuum tubes containing clot activator were allowed to clot for 20 min at room temperature prior to centrifugation at 3000 rpm for 10 min and the obtained sera were stored at −20 °C until analysis. Serum total protein concentration was measured by means of automated UV spectrophotometer (Slim, SEAC, Florence, Italy) using the Biuret method with commercially available kit (Biosystems S.A., Barcelona, Spain).

### 2.4. Serum Protein Electrophoretic Pattern Analysis

The protein standard used was bovine albumin (6.02 g/dL). Electrophoresis for protein fraction assessment was performed using an automated system (Selvet 24, Seleo Engineering, Naples, Italy) according to the procedures described by the manufacturer. A total of 25 μL of each serum sample was applied to numbered sample wells of acetate cellulose films. Each holder accommodated up to 24 samples. Films were electrophoresed for 28 min at 180 V. After electrophoresis, films were simultaneously fixed using an automated system, stained in red stain acid solution for 10 min, and then dried at 37 °C. After destaining in acetic acid and drying completely for 15 min, films were scanned on a densitometer and electrophoretic curves plus related quantitative specific protein concentrations for each sample were displayed using computer software (SelVet 24). All samples were analyzed by the same operator, who determined the lines separating fractions in the densimeter tracing [25]. The major protein fractions were divided into albumin, α1-, α-2, β- and γ- and globulins, from the cathode to the anode, according to the recommendation by the manufacturer. Relative protein concentrations within each fraction were determined as the optical absorbance percentage; then, the absolute concentration (g/dL) and albumin/globulin ratio (A/G) were calculated using the total protein concentration.

### 2.5. Statistical Analysis

The obtained data were tested for normality using the Shapiro–Wilk test. The resulting data was normally distributed (*p* > 0.05) and one-way analysis of variance (ANOVA) was applied to assess the significant influence of the administration of vaccine containing inactivated-West Nile virus on the values of the hematological parameters and on the serum protein profile of vaccinated horses. When significant differences were found, Duncan’s post hoc comparison was applied. *p* values < 0.05 were considered statistically significant.

## 3. Results

No adverse events related to the product, skin reactions or general signs were observed after vaccine injection; moreover, no change in clinical examination parameters (i.e., body temperature, nutritional status, behavioral changes, heart rate, respiratory rate depression and/or hyperthermia) was recorded.

The mean values ± standard deviation (± SD) of hematological parameters obtained from horses before and after vaccine administration are reported in Table 1. Statistical analysis showed no changes in the main hematological parameters (e.g., white blood cells, WBC; red blood cells, RBC; hemoglobin, Hb; hematocrit, Hct; mean corpuscular hemoglobin, MCH; mean corpuscular hemoglobin concentration, MCHC; mean corpuscular volume, MCV; platelets, PLT) measured in vaccinated horses compared to pre-vaccine values (*p* > 0.05). However, among the leukocyte population, statistically significant lower neutrophils values were recorded 72 h after the first WNV vaccine-dose administration (T3_I_) and before the WNV vaccine-booster (TPRE_II_) compared to the values measured at T0 (*p* < 0.01). Lymphocytes showed statistically significant higher values at T3_I_, TPRE_II_ and T1_II_ compared to T0 (*p* < 0.01). Monocytes values were statistically significantly higher after 72 h, 1week and the 2weeks from WNV vaccine-booster (T3_II_, T4_II_ and T5_II_) compared to T0 (*p* < 0.01).

Table 2 shows the mean values (± SD) of serum total proteins and their electrophoretic fractions obtained from horses before and after vaccine administration with the respective statistical significances. In particular, the Dunnet post test revealed higher serum total protein values (*p* < 0.05) throughout the post vaccine period compared to the baseline values. Moreover, the electrophoretic protein fractions showed a dynamic change following WNV vaccine administration (Table 2) with statistically significant variations after both the first vaccine-dose and vaccine-booster administration. In particular, α1-globulins showed increased values after the vaccine-booster with the highest levels measured at T3II and T4II (*p* < 0.05); α-2- and β-globulin fractions showed higher levels throughout the post-vaccine period compared to T0 (*p* < 0.05); and higher γ-globulin values were found before the vaccine-booster (TPREII) and after 24 h, 72 h and 3 weeks from the vaccine-booster administration (T1II, T3II and T6II). The A/G ratio showed statistically significant decreased values at TPREII, T1II, T3II, T4II, T5II and T6II with respect to T0 (*p* < 0.05).

## 4. Discussion

The findings obtained in the current study showed a dynamic change in some leukocyte populations including lymphocytes, monocytes and neutrophils after vaccine administration, which most probably reflects the systemic immune-biological adaptation of animals to vaccine, and particularly, to the second dose of administration. The immune stimulation after the vaccination was probably related to the proliferation of blood lymphocytes stimulated by viral proteins that are known to act as mitogens or superantigens on some lymphocyte subpopulations [26,27,28,29,30]. In comparison to the values measured in pre-vaccinated horses, an increase in lymphocytes was observed in horses after vaccination. Specifically, this rise acquired statistical significance before the vaccine-booster, coinciding with 4 weeks after the first dose administration, and 24 h after the vaccine-booster administration. Killed vaccines are considered relatively safe, because the virus cannot replicate and cause clinical disease. However, inactivated vaccines tend to have a reduced ability to induce and sustain an effective and balanced immune response, thus requiring multiple and repeated doses to maintain protection [24,25]. Furthermore, killed vaccines tend to stimulate humoral immunity more so than cell-mediated immunity. Although this humoral stimulation results in a measurable antibody titer response, it is likely that cell-mediated immunity plays a role in defence against WNV as well and is more difficult to quantify [26,27,31,32,33]. Among the leukocyte population, neutrophils and monocytes share some important features from a clinical point of view. These cells protect patients from an overwhelming risk of fatal infections. Neutrophil granules serve as reservoirs for digestive and hydrolytic enzymes prior to delivery into the phagosome [21]. Mononuclear phagocytes have at least three major functions: presentation of antigens, phagocytosis, and immunomodulation. Monocytes have Fc receptors and express the IgG receptor FcγRI (CD64) constitutively in contrast to neutrophils, which express this receptor only in response to inflammatory stimuli. Monocytes are also subject to immune modulation through the role of chemokines produced by CD8+ T cells [21]. Monocytes, macrophages, and neutrophils express a large number of cell surface proteins that play crucial functional roles in phagocyte biology. Microbial pattern-recognition receptors are an essential component of innate immunity, in which they recognize and detect pathogen-associated molecular patterns, resulting in activation of monocytes, macrophages, and dendritic cells and neutrophils as part of the host response to eradicate invading pathogens [21]. Neutrophils showed lower values 72 h after vaccine and before the vaccine-booster, whereas monocytes showed the highest values at one week and two weeks after the second administration dose. The increase in monocyte count could indicate the presence of sub-acute or chronic inflammation after the second dose of the vaccine [33]. Inflammation conditions could be reflected in a modification in the serum protein electrophoretic pattern as some protein fractions include acute phase proteins [22]. Horses affected by vector borne disease often display an increase in the concentration of total proteins, which is usually caused by dehydration and by the increase in the γ-globulin concentration [22]. Until now zone-electrophoresis to evaluate changes in serum protein fractions has not been carried out on horses vaccinated against WNV, although it is very useful from a clinical point of view because it is less time consuming. The results obtained in the current study revealed higher serum total protein values after vaccination compared to the baseline values. The increasing trend of serum total proteins was accompanied by a rise in their electrophoretic fractions. The increase in α-1 globulin may be related to the augment in the synthesis of α-1 antitrypsin, the main protein of the α-1 globulin region [34], in response to the vaccine-induced acute inflammatory process. Moreover, this antitrypsin inhibits a wide range of bacterial proteases and leukocytary enzymes [30]. In addition to α-1 antitrypsin, the α-1 globulin fraction in serum electrophoresis normally contains large amounts of other proteins such as α-1 acid glycoprotein, α-fetoprotein, transcortin and thyroxin-binding globulin. The α-2 globulin region includes serum amyloid A, haptoglobin, α-2 macroglobulin and ceruloplasmin, and the increase observed in this fraction suggests that serum amyloid A, haptoglobin and ceruloplasmin levels were augmented, following acute inflammatory response pattern [29]. α-2 macroglobulin belongs to the protease inhibitor group; notwithstanding, its clinical importance is limited [35]. Additionally, the increase in β-globulins observed in horses following vaccination indicate the development of acute inflammation. The findings obtained in the current survey are in line with previous studies carried out on vaccinated animals. Overall, it has been found that, regardless of the type of vaccine and the pathogen against which the animal is to be immunized, the vaccination induces a significant inflammatory response leading to increases in acute phase protein levels and leukocytes change [16]. In particular, it has been showed that horses vaccinated against influenza virus stains and tetanus toxoid displayed an inflammation response comparable to horses with highly inflammatory arthritis induced by intra-articular injection of lipopolysaccharide [36] or horses undergoing surgery [37]. Thus, vaccination seems to be quite a potent inducer of the acute phase response even in the absence of systemic clinical signs of inflammation, such as fever. However, changes in levels of the measured acute phase reactants were not as severe as those previously demonstrated in horses with bacterial infection [38,39]. Notably, the γ-globulin fraction showed an increasing trend throughout post-vaccine time points although the highest values were observed before the vaccine-booster, coinciding with 4 weeks after first administration, and after 24 h, 72 h and 3 weeks from the vaccine-booster. This trend seems to suggest the occurrence of immunoglobulin production, mainly IgG, IgM and IgA, a response expected with vaccination [22]. The progressive increase of the γ-globulin fraction associated with the increase of lymphocytes herein observed could suggest that the lymphocytosis observed following vaccination could mainly represented by an augment of B lymphocytes and, thus, antibody production due to antigenic stimulation [22,40]. It has been demonstrated that horses vaccinated by using a killed vaccine protocol against WNV develop a neutralizing antibody at 4–6 week after vaccination [41]. In a similar study of llamas and alpacas, more than 90% seroconverted by three weeks after the second vaccination, and more than 97% by three weeks after a third vaccination [42].

Despite the interesting results obtained, the study has limitations: only ten horses were included in this study, all geldings were aged 8–11 years, therefore, it has not possible to conclude vaccine safety, either the expected response in much younger or older horses. Furthermore, antibody levels were not determined, and PLT count was only based on automatic count therefore a PLT auto-activation has not been evaluated.

## 5. Conclusions

The current study allowed us to conclude that vaccine use does not alter the overall hemogram picture of horses although it is associated with modulation in leukocyte populations and serum protein electrophoretic patterns. Though the gathered results brought new insights on the assessment of WNV vaccine response, further studies on this topic are warranted to better evaluate the cellular response and to characterize the antibody production in order to have a more complete picture of the animal’s response to WNV vaccination.

## Figures and Tables

**Table 1:** Mean values ± standard deviation (± SD) of red blood cells (RBCs), hemoglobin (Hb), hematocrit (Hct), mean corpuscular volume (MCV), mean corpuscular hemoglobin (MCH), mean corpuscular hemoglobin concentration (MCHC), white blood cells (WBCs) together with the leukocyte population percentages, and platelets (PLTs) measured in horses before (T0), and after 24 h, 48 h, 72 h, 1 week, 2 weeks and 3 weeks (T1_I_,T2_I_, T3_I_, T4_I_, T5_I_ and T6_I_) from the first WNV vaccine-dose administration as well as before (TPRE_II_), and after 24 h, 48 h, 72 h, 1 week, 2 weeks and 3 weeks (T1_I I_,T2_II_, T3_II_, T4_II_, T5_II_, T6_II_) from the WNV vaccine-booster.

Parameters	First WNV Vaccine-Dose							WNV Vaccine-Booster						
	T0	T1_I_	T2_I_	T3_I_	T4_I_	T5_I_	T6_I_	TPRE_II_	T1_II_	T2_II_	T3_II_	T4_II_	T5_II_	T6_II_
RBCs (×10^6^/µL)	6.9 ± 0.4	6.8 ± 0.3	6.8 ± 0.4	6.8 ± 0.3	7.0 ± 0.5	7.1 ± 0.2	6.9 ± 0.3	7.0 ± 0.7	6.6 ± 0.6	6.6 ± 0.4	6.8 ± 0.7	6.9 ± 0.7	6.7 ± 0.5	6.6 ± 0.6
Hb (g/dL)	12.9 ± 1.1	12.5 ± 1.1	12.6 ± 1.0	12.3 ± 0.8	13.4 ± 0.5	13.2 ± 0.7	12.5 ± 0.9	13.2 ± 1.5	12.4 ± 0.6	12.0 ± 0.5	12.1 ± 0.6	12.0 ± 1.3	12.3 ± 1.0	12.1 ± 0.7
Hct (%)	32.5 ± 1.4	31.7 ± 1.5	32.6 ± 1.6	32.3 ± 1.6	33.6 ± 2.1	33.9 ± 1.2	31.2 ± 1.5	32.9 ± 3.4	30.5 ± 1.9	30.3 ± 1.0	30.8 ± 1.6	32.0 ± 2.9	31.6 ± 1.5	31.0 ± 1.6
MCV (fL)	47.3 ± 1.5	47.4 ± 1.8	48.2 ± 1.5	47.6 ± 1.2	47.4 ± 1.0	47.5 ± 1.1	46.5 ± 1.1	46.8 ± 1.6	46.3 ± 1.2	46.3 ± 1.6	46.0 ± 1.3	46.4 ± 1.4	46.4 ± 1.6	46.2 ± 1.6
MCH (pg)	18.7 ± 1.0	18.3 ± 1.0	18.7 ± 0.9	18.1 ± 0.4	19.2 ± 0.7	18.4 ± 0.7	18.5 ± 0.8	18.8 ± 0.9	18.0 ± 0.7	18.1 ± 0.9	18.2 ± 0.7	17.2 ± 0.7	17.9 ± 1.4	17.6 ± 1.7
MCHC (%)	39.6 ± 1.7	38.3 ± 1.0	38.6 ± 1.6	37.9 ± 0.6	40.3 ± 1.3	38.5 ± 0.6	39.8 ± 0.9	40.3 ± 1.5	38.8 ± 1.3	39.1 ± 1.7	38.7 ± 0.8	37.3 ± 0.7	38.7 ± 2.8	37.4 ± 4.9
WBCs (×10^3^/µL)	5.5 ± 0.6	6.4 ± 0.9	6.1 ± 1.0	5.3 ± 0.7	5.7 ± 0.6	5.6 ± 0.7	4.8 ± 0.6	5.7 ± 1.1	5.7 ± 1.2	5.3 ± 1.1	5.2 ± 1.0	4.9 ± 0.9	5.1 ± 0.9	5.0 ± 0.9
Lymphocytes (%)	28 ± 6	38 ± 7	35 ± 8	39 ± 6 *	37 ± 4	30 ± 5	28 ± 5	46 ± 14 *	39 ± 6 *	32 ± 9	31 ± 6	35 ± 7	32 ± 8	32 ± 6
Neutrophils) (%)	67 ± 5	68 ± 7	63 ± 8	58 ± 6 *	62 ± 4	67 ± 5	69 ± 7	52 ± 12 *	59 ± 5	64 ± 7	63 ± 7	60 ± 8	61 ± 7	64 ± 6
Monocytes (%)	2 ± 1	1 ± 1	1 ± 1	2 ± 1	1 ± 1	2 ± 1	1 ± 1	1 ± 1	1 ± 1	2 ± 1	3 ± 2 *	3 ± 2 *	4 ± 2 *	2 ± 1
Eosinophils) (%)	1 ± 1	1 ± 1	1 ± 1	1 ± 1	1 ± 0	2 ± 1	1 ± 1	1 ± 1	0 ± 0	2 ± 1	3 ± 1	2 ± 2	3 ± 3	2 ± 1
Basophils (%)	1 ± 1	1 ± 1	1 ± 1	1 ± 1	0 ± 0	1 ± 1	1 ± 1	0 ± 0	1 ± 1	1 ± 1	1 ± 1	0 ± 0	1 ± 1	0 ± 0
PLTs (×10^3^/µL)	131.8 ± 22	127.6 ± 23	136.9 ± 33	128.3 ± 17	123.7 ± 13	132.3 ± 17	122.3 ± 15	126.8 ± 19	121.1 ± 14	120.1 ± 14	119.6 ± 21	129.6 ± 21	118.3 ± 16	125.2 ± 13

* Statistically significant different from baseline values (T0, *p* < 0.01).

**Table 2:** Mean values ± standard deviation (± SD) of serum total proteins (TP) and their electrophoretic fractions (e.g., albumin, α1-, α-2, β- and γ-globulins) and A/G ratio measured in horses before (T0), and after 24 h, 48 h, 72 h, 1 week, 2 weeks and 3 weeks (T1_I_,T2_I_, T3_I_, T4_I_, T5_I_ and T6_I_) from the first WNV vaccine-dose administration, before (TPRE_II_), and after 24 h, 48 h, 72 h, 1 week, 2 weeks and 3 weeks (T1_I I_,T2_II_, T3_II_, T4_II_, T5_II_, T6_II_) from the WNV vaccine-booster.

Serum Parameters (g/dL)	First WNV Vaccine-Dose							WNV Vaccine-Booster						
	T0	T1_I_	T2_I_	T3_I_	T4_I_	T5_I_	T6_I_	TPRE_II_	T1_II_	T2_II_	T3_II_	T4_II_	T5_II_	T6_II_
Total proteins	5.4 ± 0.5	6.5 ± 0.4 *	6.6 ± 0.4 *	6.6 ± 0.4 *	6.5 ± 0.4 *	6.5 ± 0.5 *	6.6 ± 0.3 *	8.5 ± 0.1 *	6.9 ± 0.3 *	6.3 ± 0.2 *	6.4 ± 0.3 *	6.5 ± 0.2 *	6.3 ± 0.3 *	6.7 ± 0.4 *
Albumin	2.3 ± 0.4	2.8 ± 0.4	2.8 ± 0.4	2.7 ± 0.5	2.7 ± 0.4	2.8 ± 0.4	2.7 ± 0.3	2.9 ± 0.1	2.3 ± 0.1	2.7 ± 0.3	2.2 ± 0.2	2.5 ± 0.3	2.3 ± 0.4	2.3 ± 0.3
α1-globulins	0.2 ± 0.05	0.2 ± 0.06	0.2 ± 0.04	0.2 ± 0.05	0.2 ± 0.05	0.2 ± 0.06	0.2 ± 0.05	0.2 ± 0.04	0.2 ± 0.06	0.2 ± 0.03	0.3 ± 0.04 *	0.3 ± 0.04 *	0.2 ± 0.06	0.2 ± 0.05
α2-globulins	0.6 ± 0.09	0.9 ± 0.04 *	0.9 ± 0.04 *	0.9 ± 0.05 *	0.8 ± 0.1 *	0.7 ± 0.05 *	0.8 ± 0.08 *	1.2 ± 0.08 *	0.9 ± 0.04as *	0.8 ± 0.04 *	0.9 ± 0.04 *	0.8 ± 0.04 *	0.9 ± 0.1 *	1.1 ± 0.3 *
β-globulins	0.9 ± 0.1	1.3 ± 0.2 *	1.2 ± 0.1 *	1.3 ± 0.1 *	1.1 ± 0.05 *	1.1 ± 0.07 *	1.1 ± 0.07 *	1.3 ± 0.07 *	1.1 ± 0.04 *	1.1 ± 0.1 *	1.2 ± 0.09 *	1.1 ± 0.1 *	1.1 ± 0.1 *	1.2 ± 0.2 *
γ-globulins	1.4 ± 0.2	1.4 ± 0.3	1.4 ± 0.3	1.5 ± 0.3	1.7 ± 0.4	1.7 ± 0.5	1.7 ± 0.4	2.9 ± 0.2 *	2.4 ± 0.2 *	1.6 ± 0.2	1.9 ± 0.4 *	1.7 ± 0.5	1.8 ± 0.4	1.9 ± 0.3 *
A/G ratio	0.74 ± 0.1	0.74 ± 0.2	0.74 ± 0.2	0.72 ± 0.2	0.70 ± 0.2	0.75 ± 0.2	0.69 ± 0.1	0.55 ± 0.02 *	0.49 ± 0.04 *	0.75 ± 0.1	0.52 ± 0.1 *	0.56 ± 0.1 *	0.58 ± 0.1 *	0.53 ± 0.1 *

* Statistically significant different from baseline values (T0, *p* < 0.05).

## Data Availability

The data presented in this study are available on request from the corresponding author.
